# Identification of thresholds for dichotomizing DNA methylation data

**DOI:** 10.1186/1687-4153-2013-8

**Published:** 2013-06-06

**Authors:** Yihua Liu, Yuan Ji, Peng Qiu

**Affiliations:** 1 Department of Bioinformatics and Computational Biology, University of Texas MD Anderson Cancer Center, Houston, TX 77030, USA; 2 Center for Clinical and Research Informatics, NorthShore University HealthSystem, Chicago, IL 60201, USA

## Abstract

DNA methylation plays an important role in many biological processes by regulating gene expression. It is commonly accepted that turning on the DNA methylation leads to silencing of the expression of the corresponding genes. While methylation is often described as a binary on-off signal, it is typically measured using beta values derived from either microarray or sequencing technologies, which takes continuous values between 0 and 1. If we would like to interpret methylation in a binary fashion, appropriate thresholds are needed to dichotomize the continuous measurements. In this paper, we use data from The Cancer Genome Atlas project. For a total of 992 samples across five cancer types, both methylation and gene expression data are available. A bivariate extension of the StepMiner algorithm is used to identify thresholds for dichotomizing both methylation and expression data. Hypergeometric test is applied to identify CpG sites whose methylation status is significantly associated to silencing of the expression of their corresponding genes. The test is performed on either all five cancer types together or individual cancer types separately. We notice that the appropriate thresholds vary across different CpG sites. In addition, the negative association between methylation and expression is highly tissue specific.

## Introduction

DNA methylation plays an important role in cancer through hypermethylation to turn off tumor suppressors and hypomethylation to activate oncogenes [1,2]. It is widely accepted that DNA methylation is associated with silencing of gene expression [3]. With data from high-throughput array and sequencing technologies, several studies have analyzed the relationship between methylation and gene expression [4-6].

When the relationship between methylation and gene expression is discussed, both are often described as binary signals (i.e., on-off, high-low) [7]. For example, for a gene whose expression can be controlled by the methylation of a CpG site in its promoter region: if the CpG site is methylated, the gene’s expression is typically low; if the CpG site is unmethylated, the expression of the gene can be either high or low, depending on other controlling mechanisms. On the other hand, measurements of methylation and expression obtained using microarrays and sequencing technologies are in continuous values. If we want to interpret the relationship between methylation and gene expression data using the binary language, appropriate thresholds are needed to dichotomize the measurements.

To jointly analyze methylation and gene expression, an ideal dataset would be a large collection of samples for which both data types are available. The Cancer Genome Atlas (TCGA) project provides such data for a large number of cancer samples [8-11]. Moreover, the TCGA samples are derived from multiple cancer and tissue types. The diversity among the samples may enable us to see relationships that cannot be observed in individual tissue types.

In this paper, we downloaded DNA methylation and gene expression data in TCGA. Data for a total of 992 samples were available, covering five cancer types. We extended the StepMiner algorithm [12] to identify thresholds to dichotomize methylation and expression measurements. Hypergeometric test was used to identify CpG sites whose methylation is significantly associated to silencing of expression of their corresponding genes. We observed that appropriate thresholds are highly CpG site specific, and the methylation-expression association for many genes is tissue-type specific.

## Materials and methods

### Methylation and expression data from TCGA

TCGA data portal (https://tcga-data.nci.nih.gov/tcga/tcgaDownload.jsp) provides three ways for accessing the data. Two of them, ‘data matrix’ and ‘bulk download,’ require investigators to manually select a subset of the data and then automatically collect relevant data files into a compressed.tar file for download. After that, additional effort is needed to parse and assemble the downloaded files into formats useful to programming environments such as Matlab or R. Since TCGA data keep growing and the manual selection can be tedious when multiple data types and disease types are considered, it is difficult to keep track of the manual selections and guarantee reproducibility. Therefore, we chose the third way, ‘open-access http directory,’ which contains links for all individual data files in TCGA (http://tcga-data.nci.nih.gov/tcgafiles/ftp\_auth/distro\_ftpusers/anonymous/tumor/). We created Matlab scripts to programmatically grab methylation and RNA-seq data files for each individual disease type, automatically parse them, and organize them into tab delimited spreadsheets for subsequent analysis. Our scripts for automatically downloading TCGA data are available at http://odin.mdacc.tmc.edu/~pqiu/software/DownloadTCGA/.

Genome-wide methylation measurements were generated using the Illumina Infinium Human DNA Methylation27 array platform (Illumina, Inc., San Diego, CA, USA), which interrogates the methylation status of 27,578 CpG sites in proximal promoter regions of 14,475 genes in the human genome. As of 12 February 2013, methylation data for 2,796 samples across 12 cancer types were available. We downloaded the TCGA level 3 preprocessed data, which are the ratio *M*_*i*_/(*U*_*i*_+*M*_*i*_) for each CpG site *i*. *M*_i_ represents the signal intensity of the methylated probe for CpG site *i*, and *U*_i_ is the signal intensity of the unmethylated probe. Therefore, the numerical range of the data is between 0 and 1. Zero (0) indicates unmethylated, whereas 1 indicates completely methylated. The data contain a small fraction of empty entries, because the corresponding probes either overlap with known single-nucleotide polymorphisms or other genomic variations, or their signal intensities are lower than the background.

TCGA uses several platforms to quantify gene expression, among which the Illumina GA II and HiSeq platforms profiled the largest number of samples. As of 12 February 2013, preprocessed RNA-seq data for 4,108 samples across 11 cancer types were available. The preprocessed data are the RPKM values for 20,532 genes in each sample. Roughly, the numerical range of the data is between 0 and 10^5^. For each gene, we replaced the zero entries with the minimal non-zero value of this gene across all samples and transformed the data to log scale.

The total number of overlapping samples between the above methylation and expression data was 992. The overlap covered five different cancer types: breast cancer (BRCA, 313 samples), colon and rectal cancer (COAD/READ, 227 samples), kidney renal clear cell carcinoma (KIRC, 208 samples), squamous cell lung cancer (LUSC, 129 samples), and uterine corpus endometrioid carcinoma (UCEC, 115 samples). Our analysis was performed based on these 992 overlapping samples.

### Extend StepMiner for dichotomizing methylation and expression data

**Figure 1:**
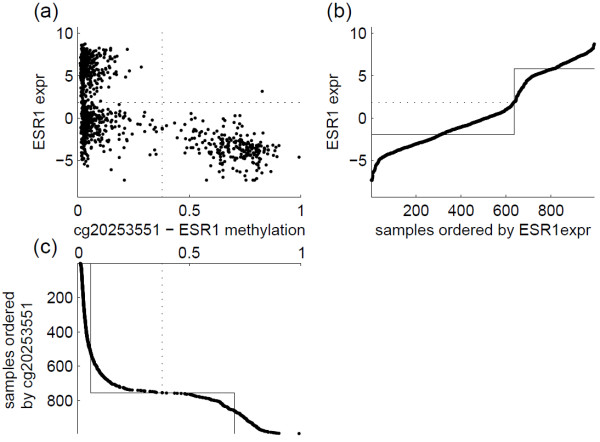
**Two examples of univariate StepMiner.** (**a**) Scatter plot of ESR1’s gene expression and methylation (cg20253551). The dotted lines indicate thresholds identified by StepMiner. (**b**) Samples are ordered according to ESR1 expression, and a step function is fitted to the ordered expression data and identify a threshold to dichotomize expression. (**c**) A step function is fitted to the ordered methylation data and identify a threshold to binarize methylation.

StepMiner was originally developed to extract binary patterns in microarray gene expression data [13] and study the boolean implications between expression of pairs of genes [12]. StepMiner examines data in a univariate fashion. Given a random variable *X* with an unknown probability distribution and *n* independent observations of the random variable *x*_*k*_,_(*k*=1,2,...,*n*)_, the algorithm first sorts the observations in ascending order *x*_(1)_≤*x*_(2)_≤...≤*x*_(*n*)_. Then, the sorted data are fitted by a step function, *f*(*i*)=*μ*_1_*I*(*i*≤*t*)+*μ*_2_*I*(*i*>*t*), where *i*=1,2,...,*n* and *I*(.) is an indicator function. Denote the mean of all observations as *μ*, the deviation of the fitted step function to sample mean as signal $=\sum_{i=1}^{n}{\left(f(i)-\mu \right)}^{2}$, and the fitting error as noise $=\sum_{i=1}^{n}{\left(f(i)-x_{\left(i\right)}\right)}^{2}$. The goodness of fit can be defined by a signal-to-noise ratio (SNR), and the best fit parameters can be found by maximizing SNR $=\frac{\text{signal}}{\text{noise}}$. Operationally, the maximization problem can be solved by exhaustively enumerating all possible integer values for 1≤*t*<*n*. For each possible value of *t*, calculating the ratio is straight forward because *μ*_1_ is the mean of the first *t* observations in the sorted data and *μ*_2_ is the mean of the remaining observations. Once the *t*^∗^ that maximizes SNR is identified, the threshold for dichotomizing the observation can be defined as $\frac{1}{2}(x_{\left(t^{*}\right)}+x_{\left(t^{*}+1\right)})$. The maximum value of signal-to-noise ratio SNR (*t*^∗^) depends on the distribution of *X*. If *X* has an extreme bi-modal distribution and its probability density function is a sum of two delta functions, SNR (*t*^∗^) approaches positive infinity. If *X* follows a uniform distribution, SNR (*t*^∗^) equals to 3. If *X* follows a Gaussian distribution, SNR (*t*^∗^) is approximately 1.75, regardless of the values of mean and variance of the Gaussian. Two examples using real data are shown in Figure 1, illustrating how univariate StepMiner identifies thresholds for gene expression of ESR1, and cg20253551 which measures the methylation status of a CpG site of that gene.

As shown above, StepMiner is a univariate algorithm, which determines a threshold for each feature by its marginal distribution. Since we are interested in the relationship between the expression of a gene and its methylation, one natural idea is to extend the algorithm to a bivariate analysis and jointly consider two variables, which we call StepMiner2D. As shown in Figure 1b, the univariate StepMiner assigns each observation to a point, whose *x*-coordinate is the rank of this observation, and *y*-coordinate is the observed value itself. The collection of all observations forms a non-decreasing curve which is fitted by a step function. In order to extend the algorithm to bivariate, we define a non-decreasing surface and fit it with a bivariate step function. Given *n* observations of two random variable *X* and *Y*, (*x*_*k*_,*y*_*k*_),_(*k*=1,2,...,*n*)_, we assign each observation (*x*_*k*_,*y*_*k*_) to a point in a three-dimensional (3D) space. The *x*-coordinate of the point is the rank of *x*_*k*_ with respect to all the observations for *X*; the *y*-coordinate of the point is the rank of *y*_*k*_ with respect to all the observations for *Y*; and the *z*-coordinate of the point is *x*_*k*_+*y*_*k*_. The collection of all points forms a surface, which is fitted by a bivariate step function with six parameters,

(1) $$\begin{array}{rl} f(i,j)= & \mu_{11}I(i\leq t_{x},j\leq t_{y})+\mu_{12}I(i>t_{x},j\leq t_{y})\\ & +\mu_{21}I(i\leq t_{x},j>t_{y})+\mu_{22}I(i>t_{x},j>t_{y}), \end{array}$$

where *i* and *j* both range from 1 to *n*.

**Figure 2:**
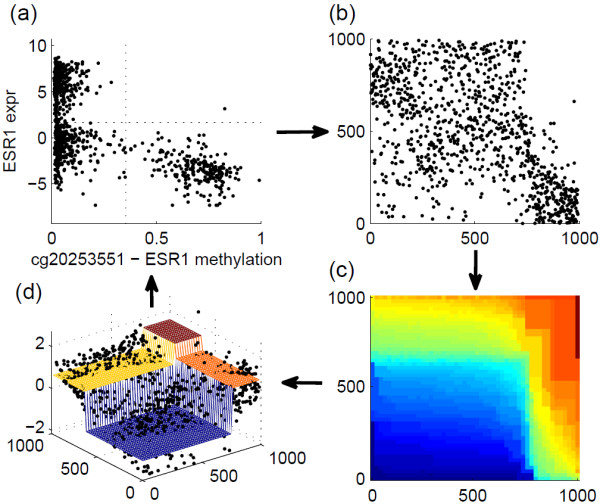
**An example of StepMiner2D.** (**a)** Scatter plot of ESR1’s gene expression and methylation. The dotted lines indicate threshold identified by StepMiner2D. (**b**) Scatter plot of rank transformed data. (**c**) Heatmap showing a non-decreasing surface sitting on top of the rank transformed data. (**d**) Three-dimensional visualization of points on the non-decreasing surface and the best fit bivariate step function.

To illustrate how to compute StepMiner2D, one example is shown in Figure 2 using the same data as the previous example. Scatter plot of ESR1’s methylation and expression is shown in Figure 2a, along with the thresholds identified by StepMiner2D. To compute the thresholds, we first perform rank transformation. Assume *x*_*k*_ is the *i*th smallest among all observed value for *X*, and *y*_*k*_ is the *j*th smallest among all observed value for *Y*, the data point (*x*_*k*_,*y*_*k*_) is mapped to point (*i*,*j*). The rank transformed data are shown in Figure 2b. To form a non-decreasing surface sitting on top of the rank transformed data, a ‘height’ *z*(*i*,*j*)=*x*_*k*_+*y*_*k*_ is defined at point (*i*,*j*) to which the *k*th observation is mapped. For an (*i*,*j*) point to which no observation is mapped, we define $z(i,j)=\underset{u\leq i,v\leq j}{\max}z(u,v)$. Such a definition guarantees that the surface is non-decreasing, i.e., $z(i,j)\geq \underset{u\leq i,v\leq j}{\max}z(u,v)$. To ensure that *X* and *Y* contribute equally, the observations are normalized to zero-mean-unit-variance before defining *z*. Finally, denote $\mu =\frac{1}{n^{2}}\sum_{i,j=1}^{n}z(i,j)$, the parameter values of the best fit two-dimensional (2D) step function can be found by optimizing an SNR $=(\sum_{i,j=1}^{n}{\left(f(i,j)-\mu \right)}^{2})/(\sum_{i,j=1}^{n}{\left(f(i,j)-z(i,j)\right)}^{2})$ in a similar exhaustive search fashion as the one-dimensional (1D) case. Computing *z* and optimizing SNR on a *n*×*n* grid can be time consuming when *n* is large. For computational efficiency, we approximate the surface on a 50×50 grid. In Figure 2c, the surface *z* is shown as a heatmap, where blue indicates small value and red indicates large value. Figure 2d shows the points (*x*_*k*_,*y*_*k*_,*z*_*k*_) and the best fit bivariate step function. The optimal SNR for this example is 5.17. Similar to the 1D case, the maximum value of SNR depends on the joint distribution of *X* and *Y*. If the joint probability density function is a sum of two or three delta functions, the optimal SNR approaches positive infinity. If *X* and *Y* are independent, the optimal SNR is 3 for uniform distribution and approximately 1.75 for Gaussian distribution.

### Hypergeometric test for methylation controlled genes

The optimal SNR value in StepMiner2D measures the multi-modality of the joint distribution of *X* and *Y*, rather than the association between the two variables. For example, if *X* and *Y* independently follow two bi-modal distributions, although there is no association between the two variables, the optimal SNR can be large. Thus, SNR does not seem to be suitable for evaluating the association between methylation and expression. Here, we are interested in one particular kind of association, whether methylation of a CpG site leads to down-regulation of its corresponding gene expression. After dichotomizing methylation and expression data, the sufficient statistics become counts of points in the four quadrants in Figure 2a. The significance of methylation controlled gene can be intuitively explained as whether the observed count in the upper-right quadrant is significantly less than expected. Popular statistical tests for 2×2 contingency tables, such as Fisher exact and chi-square tests, are designed to evaluate the whether counts are significantly unbalanced but not toward a specific direction. We choose to use hypergeometric test. Let *N* denote the total number of samples; *R* is the total number of methylated samples (sum of points in the upper-right quadrant and the lower-right quadrant); *U* is the total number of samples with high gene expression (total number of points in the two upper quadrants). Condition on *N*, *R*, and *U*, if the methylation and expression are independent, the number of samples in the upper-right quadrant *k* follows a hypergeometric distribution p(k)=UkN−UR−k/NR. To evaluate the significance of the observed count in the upper-right quadrant *K*, we can compute the probability of observing *K* or less points under the assumption that methylation and expression are independent *p* value $=\sum_{k=0}^{K}p(k)$. This is a hypergeometric test specifically for evaluating the significance of whether methylation turns off gene expression.

## Results

### Data preprocessing

We preprocessed the TCGA data by filtering out CpG sites with small variance or many missing data points and matching methylation and expression data according to genes. The methylation data we downloaded from TCGA were generated by the Methylation27 array platform, which provided the methylation status of 27,578 CpG sites in 14,475 genes across 992 cancer samples. We excluded CpG sites whose annotated genes are not present in the expression data. We also excluded CpG sites with more than 1% missing data and ones whose methylation beta value is smaller than 0.01 for more than 95% of the samples. After applying these filtering criteria, we obtained a total of 11,189 CpG sites annotated to 7,344 unique genes. For approximately half of the genes, only one CpG site is measured for each gene; data for two CpG sites are available for the majority of the other half; for a very small number of genes, measurements of multiple CpG sites are available. In the subsequent subsections, for the methylation data of each of the 11,189 CpG sites, we extracted the expression data of its corresponding gene and focused our bivariate analysis on features paired according to genes. Preprocessed data and the code for our analysis is available at http://odin.mdacc.tmc.edu/~pqiu/projects/MethExpr/.

### Identification of methylation on-off threshold

**Figure 3:**
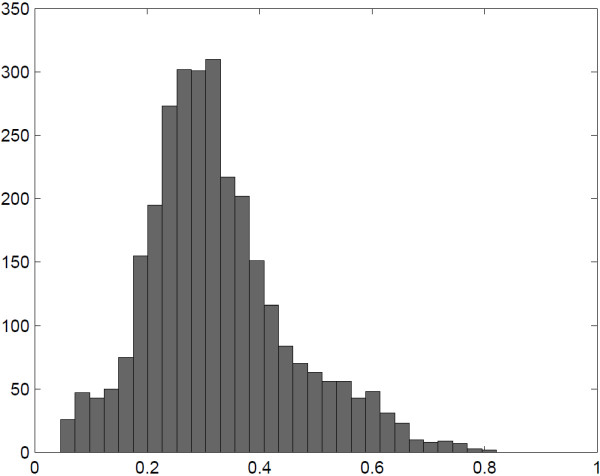
**Histogram of thresholds for dichotomizing methylation data.** For the 2,976 significant methylation-expression associations, StepMiner2D identified 2,976 thresholds for dichotomizing the methylation data. A histogram of those thresholds shows that the appropriate value for binarizing methylation varies for different methylation sites.

For each of the 11,189 methylation-expression pairs, we applied StepMiner2D to examine the data for all 992 samples together. Using such a pan-cancer analysis strategy, we identified thresholds to dichotomize the data. We performed hypergeometric test to examine whether methylation was significantly associated to the down-regulation of expression of its corresponding gene. We filtered out cases where the number of samples in the upper-right quadrant minus that in the lower-left quadrant was more than 10% of the total number of samples, which obviously did not support the concept of methylation turning off the expression. Using a *p* value threshold of 0.01 and Bonferroni correction, 2,976 pairs showed significant association, and the ESR1 example in Figures 1 and 2 was among the significant ones. Figure 3 shows a histogram of the identified methylation thresholds for the 2,976 significant associations, where we observed a wide-spread distribution. This result indicates that although the beta value quantification of methylation has a consistent numerical range of [0, 1] across different genes, the appropriate threshold for dichotomizing the beta values is highly CpG site specific.

**Figure 4:**
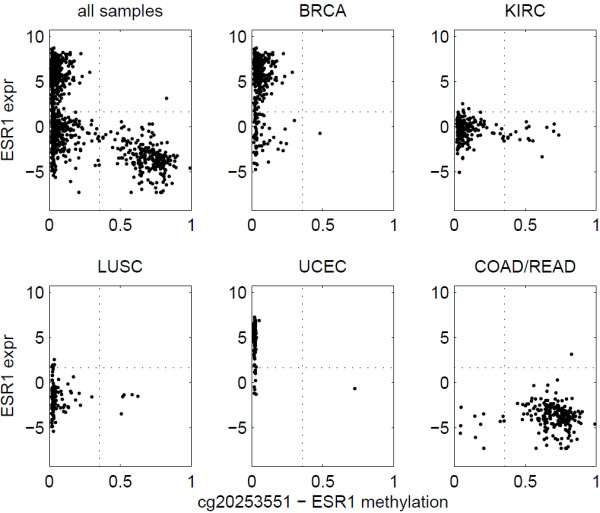
**ESR1 methylation and expression.** Scatter plots of methylation and expression data for ESR1 in all five cancer types together and individual cancer types separately. The dotted lines indicate the thresholds identified by StepMiner2D using all cancer types together. The association is significant when all cancer types are examined together but insignificant in individual cancer types.

The association between methylation and expression of ESR1 was significant when all 992 samples were examined together. However, when we examined individual cancer types separately, the methylation-expression association for ESR1 became insignificant. In Figure 4, the thresholds in all the plots are the same, and they were derived by considering all cancer types together. If we focus on breast cancer samples and ignore the rest, we see that almost all breast cancer samples are ESR1 unmethylated, and their ESR1 expression can be either high or low, which does not contradict with the concept that methylation turns off the expression. However, since very few breast cancer samples are methylated, we do not know whether methylated samples will have high ESR1 expression or low expression. Thus, we do not have strong evidence that ESR1 methylation turns off its expression in breast cancer samples, because we do not have enough points in the upper-right and lower-right quadrants. In this case, the lack of ESR1 methylated samples makes it impossible to prove the association between ESR1’s methylation and expression in breast cancer. Similarly, for COAD/READ, although most samples exhibit high methylation and low expression, the association is also insignificant. Since ESR1 is seldom highly expressed in either methylated or unmethylated samples of COAD/READ, there is little evidence that the low expression of ESR1 in COAD/READ is caused by methylation or some other regulatory mechanisms. Similar observations can be made for other cancer types in Figure 4. In fact, if the StepMiner2D method is applied to individual cancer types separately, we will not be able to identify the appropriate thresholds for dichotomizing the methylation data. This observation illustrates the power of the pan-cancer analysis strategy that includes multiple cancer types. However, this also raises a question. Maybe the observed ESR1 methylation-expression association is simply a statistical property induced by tissue differences, rather than an indication of a real mechanistic interaction. In the next subsection, we will discuss this issue further.

### Tissue-specific association between methylation and expression

**Figure 5:**
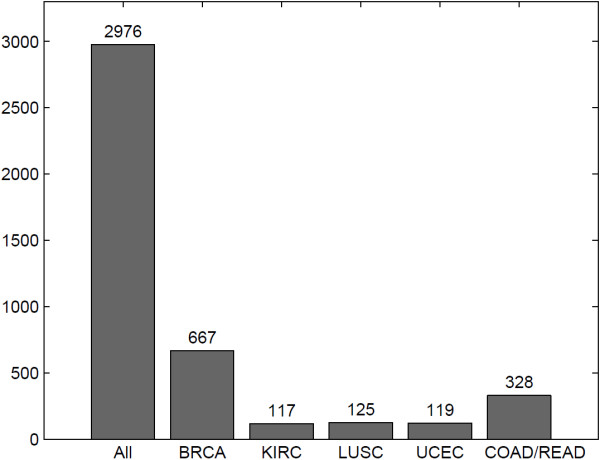
**Methylation-expression association is tissue specific.** The bar plot shows the number of significant associations when all cancer types are considered together and the numbers when individual cancer types are considered separately.

**Figure 6:**
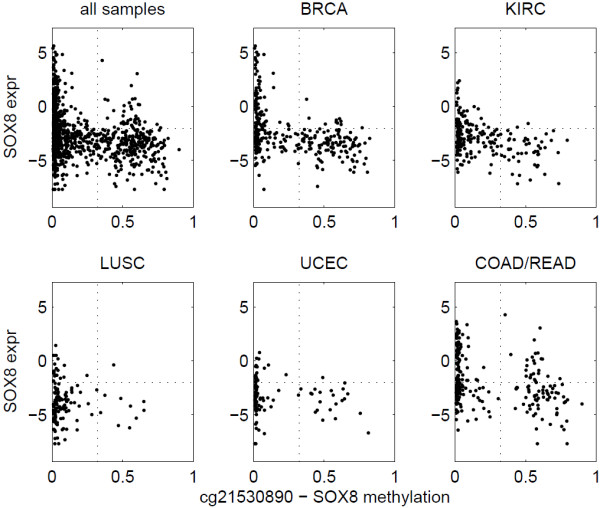
**SOX8 methylation and expression.** The methylation-expression association for SOX8 is significant in BRCA and KIRC but not in the other three cancer types.

**Figure 7:**
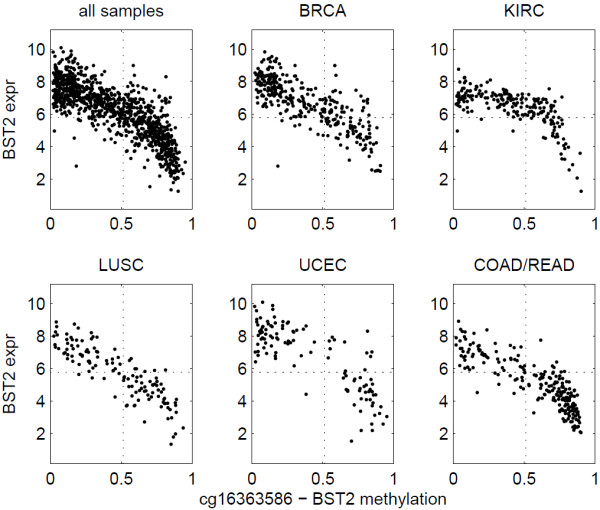
**BST2 methylation and expression.** The methylation-expression association for BST2 is significant in all five cancer types examined here.

We evaluated the association between methylation and expression using samples in individual cancer types separately. Figure 5 shows the number of significant methylation-expression associations, when all cancer types were examined together and separately. One hundred eleven insignificant associations in pan-cancer analysis turned out to be significant in an individual cancer type. Among all the 2,976 significant associations in pan-cancer analysis, 2,072 were insignificant in all five individual cancer types, similar to the pattern shown in Figure 4. The majority of the remaining associations were significant in only one or two cancer types, indicating that the association between methylation and expression is highly tissue specific. For example, Figure 6 shows that the methylation of SOX8 is significantly associated to low SOX8 expression in breast cancer and kidney renal clear cell carcinoma but not in the other three cancer types. Such tissue-specific relationship echoes a previous result that hierarchical clustering of methylation data is able to separate tissue types and cancer subtypes [14,15]. Four genes showed significant methylation-expression association in all five individual cancer types, BST2, SLA2, GSTT1, and GSTM1. Figure 7 shows the data for BST2. We think that genes showing significant association in at least one individual cancer type are more likely to represent mechanistic methylation-expression interactions, compared to the ones that are only significant when all cancer types are considered.

## Conclusions

We performed integrative analysis of methylation and gene expression data of five cancer types in TCGA. First, we pooled samples from all five cancer types together and applied StepMiner2D to identify thresholds for dichotomizing the methylation and expression data. In such a pan-cancer analysis strategy, the diversity and variation among samples allow us to observe positive and negative signals in sufficient number of samples and empower the method to identify the appropriate thresholds. Then, we applied hypergeometric test to identify CpG sites whose methylation is significantly associated to silencing of the expression of their corresponding genes, either using all five cancer types together or using individual cancer types separately. When all five cancer types were examined together, 2,976 CpG sites showed significant negative association with gene expression. However, when samples in different cancer types were considered separately, a much smaller number of significant associations were observed in at least one cancer type. We speculate that the associations only significant in pan-cancer analysis are likely to be induced by tissue differences, whereas significant associations observed in individual cancer types are more likely to reflect regulatory relationships between methylation and gene expression. For future work, there are a few possible extensions. The methylation data used here are generated by the Illumina Methylation 27k platform. TCGA also generates methylation data using the Illumina Methylation 450k platform, which measures roughly 20 times more CpG sites. We plan to redo the analysis using the 450k methylation data, which will enable us to identify more associations between methylation and expression. Moreover, the proposed analysis strategy can also be applied to examine associations among measurements made by other modalities, such as microRNA expression, DNA copy number variation, protein expression, etc.

## Competing interests

The authors declare that they have no competing interests.
